# Evaluation of the potential herb-drug interaction between Bojungikki-tang and PD-L1 immunotherapy in a syngeneic mouse model

**DOI:** 10.3389/fphar.2023.1181263

**Published:** 2023-05-18

**Authors:** Sung-Yoon Yang, Jin-Mu Yi, Jaemoo Chun, Seongwon Park, Tham Thi Bui, Hwi-Yeol Yun, Jung-Woo Chae, Mi-Kyung Jeong

**Affiliations:** ^1^ College of Pharmacy, Chungnam National University, Daejeon, Republic of Korea; ^2^ KM Convergence Research Division, Korea Institute of Oriental Medicine, Daejeon, Republic of Korea

**Keywords:** PD-L1, atezolizumab, Bojungikki-tang, CMT-167, drug-drug interaction

## Abstract

Atezolizumab (a PD-L1 inhibitor) has shown remarkable efficacy and tolerability in various cancer types. Despite its efficacy and safety, atezolizumab monotherapy has limitations, such as acquired resistance and adverse events. Bojungikki-tang (BJIKT) is an herbal decoction widely prescribed in Asian countries and used to treat cancer-related symptoms including fatigue, appetite loss, gastrointestinal disorders, and other side effects from cancer therapy. Due to its immunomodulatory effects, Bojungikki-tang has been investigated as a combined treatment with anticancer agents. We evaluated the potential drug-drug interaction (DDI) between Bojungikki-tang and the anti-PD-L1 antibody based on the Food and Drug Administration (FDA) guidelines. In the study, we conducted an *in vivo* drug-drug interaction study using a syngeneic mouse model of CMT-167 in C57BL/6. We then determined the antibody concentrations to evaluate the pharmacokinetic (PK) drug-drug interaction and measured variable biomarkers related to therapeutic efficacy and immune response. The pharmacodynamic (PD) drug-drug interaction study investigated changes in response between anti-PD-L1 antibody monotherapy and combination therapy. Using the pharmacokinetic and pharmacodynamic data, we conducted a statistical analysis to assess drug-drug interaction potential. In the presence of Bojungikki-tang, the pharmacokinetic characteristics of the anti-PD-L1 antibody were not changed. This study suggested that combination treatment with Bojungikki-tang and atezolizumab is a safe treatment option for non-small cell lung cancer. Clinical studies are warranted to confirm this finding.

## 1 Introduction

In the last decade, immunotherapy has achieved remarkable advances in oncology. Immune checkpoint inhibitors (ICIs) are anti-cancer medications which reinvigorate the host immune system to attack tumor cells by targeting immune co-inhibitory receptors, such as programmed cell death 1 (PD-1) and cytotoxic T lymphocyte antigen 4 (CTLA-4), expressed on the surface of T-cells (Sadeghi Rad et al., 2021). Atezolizumab is a monoclonal antibody that targets the programmed death ligand 1 (PD-L1)/PD-1 axis, thereby inhibiting the immune escape of tumor cells and restoring T-cell responses. Although atezolizumab monotherapy resulted in reliable efficacy and acceptance in cancer patients (Nishijima et al., 2017; Shah et al., 2018; Herbst et al., 2020), only a subset of patients responded to the treatment and even those patients are at risk of acquiring resistance to atezolizumab monotherapy (Ma et al., 2019; Morganti and Curigliano, 2020). To overcome these issues, combination strategies with other ICIs and conventional chemotherapy are suggested (Shah et al., 2018; Morganti and Curigliano, 2020).

Recently, several studies have investigated the use of herbal medicines alongside anticancer drugs to alleviate chemotherapy-induced side effects and produce synergistic therapeutic effects (Hu et al., 2016; Ma et al., 2019). Bojungikki-tang (BJIKT) is a herbal medicine composed of *Ginseng Radix*, *Atractylodis Rhizoma Alba, Astragali Radix*, *Angelicae Gigantis Radix*, *Zizyphi Fructus*, *Bupleuri radix*, *Citri Unshius Pericarpium*, *Cimicifugae Rhizoma*, *Zingiberis Rhizoma Recens*, and *Glycyrrhizae Radix et Rhizoma*. It is widely used for the treatment of appetite loss (Onogi et al., 2006), fatigue (Jeong et al., 2010), and loss of vigor (Tohda et al., 2008). It has also been reported that BJIKT has immunomodulatory effects (Yang et al., 2015; Nakakubo et al., 2020). Currently, *in vitro* and *in vivo* studies have demonstrated that BJIKT plus chemotherapy yields a synergistic interaction, leading to the alleviation of side effects and inhibition of cancer progression (Jeong et al., 2010; Ouyang et al., 2014; Sato et al., 2015; Yu et al., 2017). Recent research verified that co-administration of BJIKT with the anti-PD-L1 antibody significantly improved T-cell response in the MC38 tumor-bearing mouse model, highlighting its potential therapeutic viability for cancer treatment (Chun et al., 2022).

However, potential drug-drug interaction (DDI) may occur when two or more drugs are administered concurrently. One drug (perpetrator) could alter the pharmacokinetics (PK) or desired therapeutic outcomes of the co-administered drugs (victim drugs). Therefore, potential risk assessments and clinical evaluations should be performed to understand safety and efficacy. Even though the efficacy of BJIKT has been proven in many studies, DDI potential with co-administered drugs remains untested. For the combination of BJIKT and anti-PD-L1 antibody therapy, the potential risk of DDI should also be evaluated. The Food and Drug Administration (FDA) has recently released draft guidance related to the assessment of potential DDI for therapeutic proteins (TP) (FDA, 2020). The guidance highlighted two mechanisms for potential DDI which should be considered when TP DDI evaluations are performed: 1) proinflammatory cytokine-related mechanisms, including those where the TP is a proinflammatory cytokine or a cytokine modulator, and 2) mechanisms unrelated to cytokine modulators.

We evaluated the potential DDI between BJIKT and the anti-PD-L1 antibody based on the FDA guidelines to assess the safety and efficacy of the promising combination therapy. In the current study, we first conducted an *in vivo* DDI study using a syngeneic mouse model of CMT-167 in C57BL/6. We then determined the antibody concentrations to evaluate the PK DDI, and measured variable biomarkers related to therapeutic efficacy and immune response. The PD DDI study investigated changes in pharmacodynamic (PD) response between anti-PD-L1 antibody monotherapy and the combination therapy. Using the PK and PD data, we then conducted a statistical analysis to compare the values between groups to assess DDI potential.

## 2 Materials and methods

### 2.1 Materials and cell line

#### 2.1.1 Bojungikki-tang

BJIKT is an herbal extract that consists of 10 medicinal herbs. The extract was provided by the GMP pharmaceutical factory of Hanpoong Pharmaceutical Co., Ltd., (Jeonju, Korea). BJIKT extract used in this study meets all standards of ‘Bojungiki-tang Soft∙Dry Extract’ in the Korean Herbal Pharmacopoeia (K.H.P). In brief, 10 crushed herbs were extracted using water refluxed at 95°C. Then the extract solution was filtered, evaporated under low pressure, and lyophilized in a freeze dryer. The extraction yield was approximately 14.8% (w/w). For characterization of the components in BJIKT, LC-MS/MS method was developed to determine the components in BJIKT extract in the previous study (Chun et al., 2022), indicating that 28 compounds were identified in BJIKT extract (Supplementary Figure S1; Supplementary Table S1 in Supplementary Material). The dried powder was stored in a 4°C cold room until use.

#### 2.1.2 Anti-PD-L1 antibody

Anti-hPD-L1-mIgG1 (an anti-PD-L1 antibody) is a reverse chimera and mouse IgG1 variant antibody to murine PD-L1, which features a variable region equivalent to that of atezolizumab. The antibody was generated to contain the constant region of mouse IgG1, limiting both antibody-dependent cytotoxicity and complement-dependent cytotoxicity. The antibody was purchased from Invivogen.

#### 2.1.3 Cell line

CMT-167 is a highly metastatic subclone of murine alveogenic lung carcinoma cell line CMT 64. The cell line was purchased from the European Collection of Authenticated Cell Cultures (ECACC). Reagents for culturing CMT-167 were purchased from HyClone TM. The cell line was cultured in DMEM media containing 10% FBS, 100 U/mL penicillin, and 100 μg/mL streptomycin at 37°C in an incubator supplied with 5% CO_2_.

### 2.2 Study design

#### 2.2.1 Syngeneic mouse model of CMT-167 cells in C57BL/6

All animals were housed in specific pathogen-free circumstances. Animal study was conducted in compliance with the Animal Experimental Ethics Regulations of Osong Advanced Medical Industry Promotion Foundation (KBIO-IACUC-2021-114), as accredited by Association for Assessment and Accreditation of Laboratory Animal Care International (AAALAC) and Korea Excellent Animal Testing Facility (KELAF). Six-week-old female C57BL/6 mice were injected with 1 × 10^5^ CMT-167 cells subcutaneously. The study was initiated when the mouse tumor volume reached 80–120 mm^3^.

#### 2.2.2 *In vivo* DDI study

**FIGURE 1:**
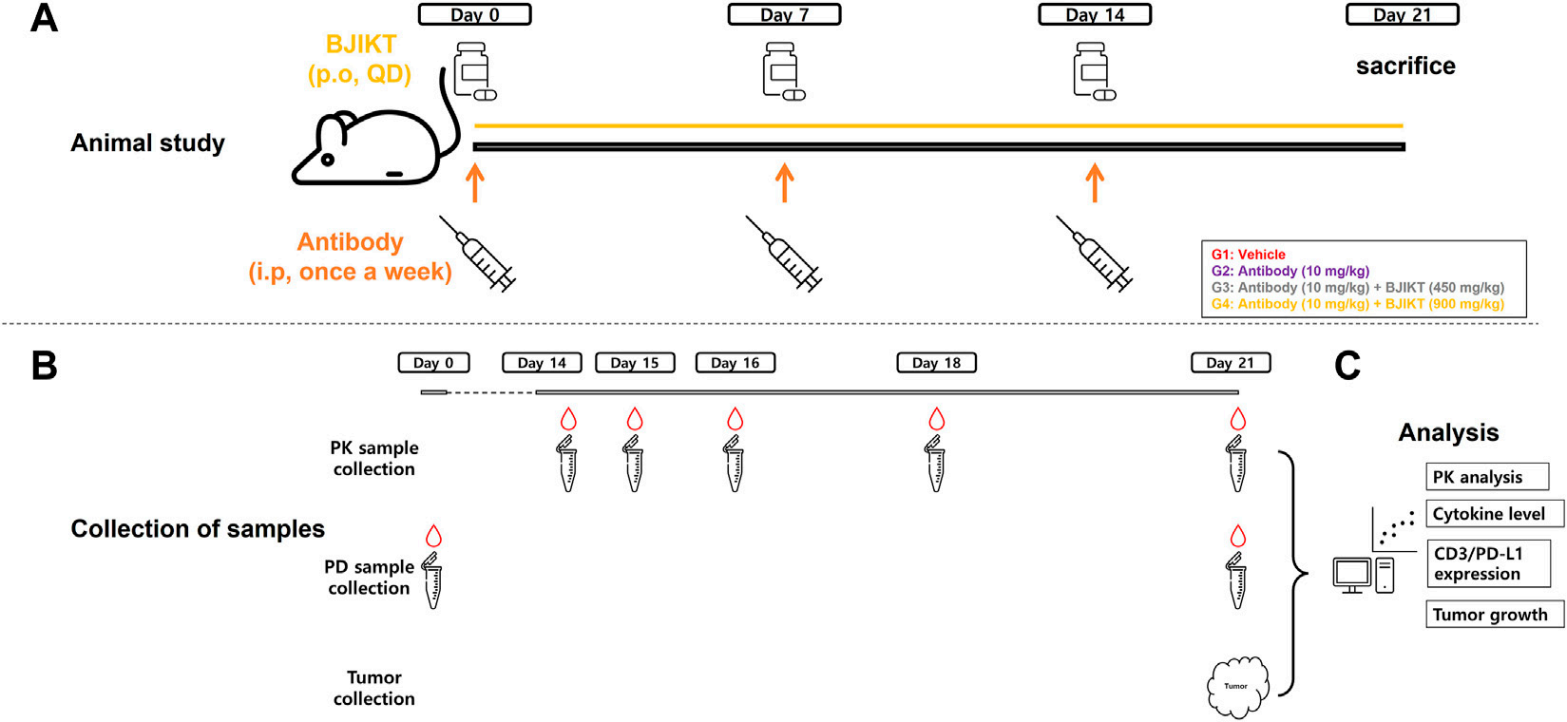
PK-PD DDI study scheme for Bojungikki-tang plus anti-PD-L1 antibody therapy. **(A)** Animal study was conducted using syngeneic mouse model of CMT-167 cells. Mice were divided into the control (G1) and test groups (G2∼G4) (*n* = 7, each group). Animals were administered with drugs during the treatment period (21 days) and sacrificed at the end of date (Day 21); **(B)** Blood samples (100 μL) were collected at the indicated time points for PK analysis and PD analysis, and tumor tissues were collected to measure tumor volume growth, CD3 cells, and PD-L1 expression; **(C)** For PK analysis, concentrations of anti-PD-L1 antibody were determined and PK parameters were calculated using NCA. Cytokine levels, CD3/PD-L1 expression levels, and tumor growths were measured for PD analysis.

Preclinical *in vivo* study was conducted to assess the drug interactions for the combination therapy (Figure 1). As a control group, group 1 (G1, *n* = 7) received saline via intraperitoneal (i.p.) injection once a week. To evaluate PK-PD DDI between BJIKT and the anti-PD-L1 antibody, test groups were established for the anti-PD-L1 antibody monotherapy and for co-therapy. With respect to the test groups, group 2 (G2, *n* = 7) received 10 mg/kg of the anti-PD-L1 antibody via i.p., injection once weekly and the co-therapy groups (G3 and G4, *n* = 7 respectively) received 10 mg/kg of the anti-PD-L1 antibody via i.p., injection once a week with co-administration of BJIKT at a dose of 450 mg/kg/day (G3) or 900 mg/kg/day (G4) using oral gavage. The optimal BJIKT dose level for the mouse study was calculated using the clinical dose of BJIKT and a dose-equivalence factor that applies to surface area between species (Nair and Jacob, 2016). All animals were weighed over the experiment period (3 weeks) after initial treatments on Day 0. At the end of the study (Day 21), the mice were sacrificed, and tumor tissues were collected.

#### 2.2.3 Sample collection

For the pharmacokinetic analysis of the antibody, blood samples (80∼100 μL) for G2, G3, and G4 were collected into serum-separating tubes (SST) at the indicated time points of 8, 24, 48, 96, and 168 h post-dose of the anti-PD-L1 antibody on Day 14. For the pharmacokinetic analysis of BJIKT, blood samples (80∼100 μL) for G4 and G5 were collected into SST at the indicated time points of 0.5, 1, 4, 8, 24 h post-dose of BJIKT on Day 14. For the analysis of serum cytokine levels, blood samples (80∼100 μl) for G1∼G4 were collected pre-antibody-dose on Day 0 and before sacrifice on Day 21.

### 2.3 Evaluation of pharmacokinetic DDI

#### 2.3.1 Assay for quantification of the anti-PD-L1 antibody in mouse serum

The analysis method of enzyme-linked immunosorbent assay (ELISA) for the an-ti-PD-L1 antibody was developed and validated to determine the serum concentrations of the antibody in mice (Supplementary Material). Aliquots of 2 μg/mL of recombinant mouse PD-L1/B7-H1 Fc chimera protein were coated on the plate to capture the PD-L1 inhibitor antibody. Calibration standard samples were prepared to set up calibration range, and quality control (QC) samples were prepared to test accuracy and precision of the developed method. The samples were accordant with acceptance criteria for validation of bio-analytical methods. All the samples were run in duplicate. The obtained data were used to calculate the PK parameters of the anti-PD-L1 antibody.

#### 2.3.2 Non-compartmental pharmacokinetic analysis

Non-compartmental pharmacokinetic analysis was performed to calculate pharmacokinetic parameters with the observed serum concentration-time profiles of anti-PD-L1 antibody and active compounds of BJIKT using R packages (Non-Compart and ncar). To evaluate PK DDI, PK parameters of anti-PD-L1 antibody were calculated in the presence/absence of BJIKT. AUCt was calculated using a linear log trapezoidal method. The half-life (t1/2) was calculated with the following equation: t1/2 = 0.693/the terminal elimination rate constant (Kel). The clearance (CL) was determined by dose given and AUCt (CL = Dose/AUCt), and the volume of distribution (Vd) was determined by CL and the Kel (Vd = CL/kel).

### 2.4 Evaluation of pharmacodynamic DDI

#### 2.4.1 Tumor volume growth

Tumor volume is used as the primary endpoint to test the efficacy of the treatment in this study. Accordingly, changes in tumor volumes were monitored throughout the study. The maximum length (L) and the perpendicular width (W) of tumor tissues were measured using a digital caliper. Tumor volumes were calculated using the formula: [Tumor volume (mm^3^) = L (mm) × W2 (mm^2^) × 1/2]. Over the treatment period, tumor volumes were measured twice weekly.

#### 2.4.2 Serum cytokine level

As a surrogate biomarker for immunotherapy, serum cytokine levels were measured to investigate PD DDI in this study (Singh et al., 2019). The temporal variance and magnitude of mouse serum cytokine levels can also identify whether the antibody is related to cytokine-inflammation mechanisms. Multiple cytokines in serum, including pro-inflammatory and anti-inflammatory cytokines, were simultaneously quantified via Bio-Plex 200 (Bio-Rad, Hercules, CA) according to the manufacturer’s manual. IL-2, IL-6, IL-10, TNF-α, and IFN-γ were selected for measurement since these cytokines are referenced as potential biomarkers correlating to pathological features in patients with non-small cell lung cancer (NSCLC) (Enewold et al., 2009; An et al., 2021). Calibration and QC samples for determining serum cytokine levels were prepared by the complete kit containing capture antibody, recombinant protein, and detection antibody. The instrument was validated with a Bio-plex validation kit within 1 week of the test and calibrated on the test date using a Bio-Plex calibration kit.

#### 2.4.3 Flow cytometric analysis

Tumor infiltrating lymphocytes (TILs) are populations of lymphocyte cells that have invaded tumor cells and played a vital role to indicate the extent of anti-tumor efficacy. PD-L1 expression on tumor surface is considered an indicator of treatment pharmacodynamic biomarkers related to the mechanism of action for the anti-PD-L1 antibody. Flow cytometry was used to measure the amount of cluster of differentiation 3 (CD3) and PD-L1 expression on tumor cell surfaces. Tumor samples were kept at −80°C until lyse, wash, and stain procedure. Enzyme mix was prepared by adding RPMI, Enzyme D, Enzyme R, and Enzyme A to a GentleMACS C tube. Mouse tumor tissues were cut into smaller pieces and dissociated using GentleMACS C tubes. The dissociated tumor cells were filtered through a 70 μM MACS smart strainer and washed with RPMI 10 mL. After 7-min centrifugation at 300 g, the cells were re-suspended with 2 mL of FACS staining buffer (PBS + 2% FBS), with each FACS tub containing 1 × 106 cell/100 μL buffer. Mouse PBMC (CD3), APC anti-mouse CD274 (PD-L1), isotype control group and experimental group antibodies were prepared for immunostaining. The percentage of CD3^+^ cells and PD-L1+ on tumor cell surface was determined by FACSCantoⅡ [Becton Dickinson and Company (BD)].

### 2.5 Statistical analysis

All statistical analyses were conducted using R (version 4.2.0). Data were expressed as mean ± standard deviation. With small sample sizes, non-parametric analytical tests were used. The Kruskal-Wallis test was used to compare the groups’ pharmacokinetic parameters, tumor volume, serum cytokine levels, CD3^+^ cells, and PD-L1 expression. The Mann-Whitney U test was used to compare the values mentioned above between the two groups. *p*-values of less than 0.05 were considered statistically significant.

## 3 Results

### 3.1 Preclinical *in vivo* study

**FIGURE 2:**
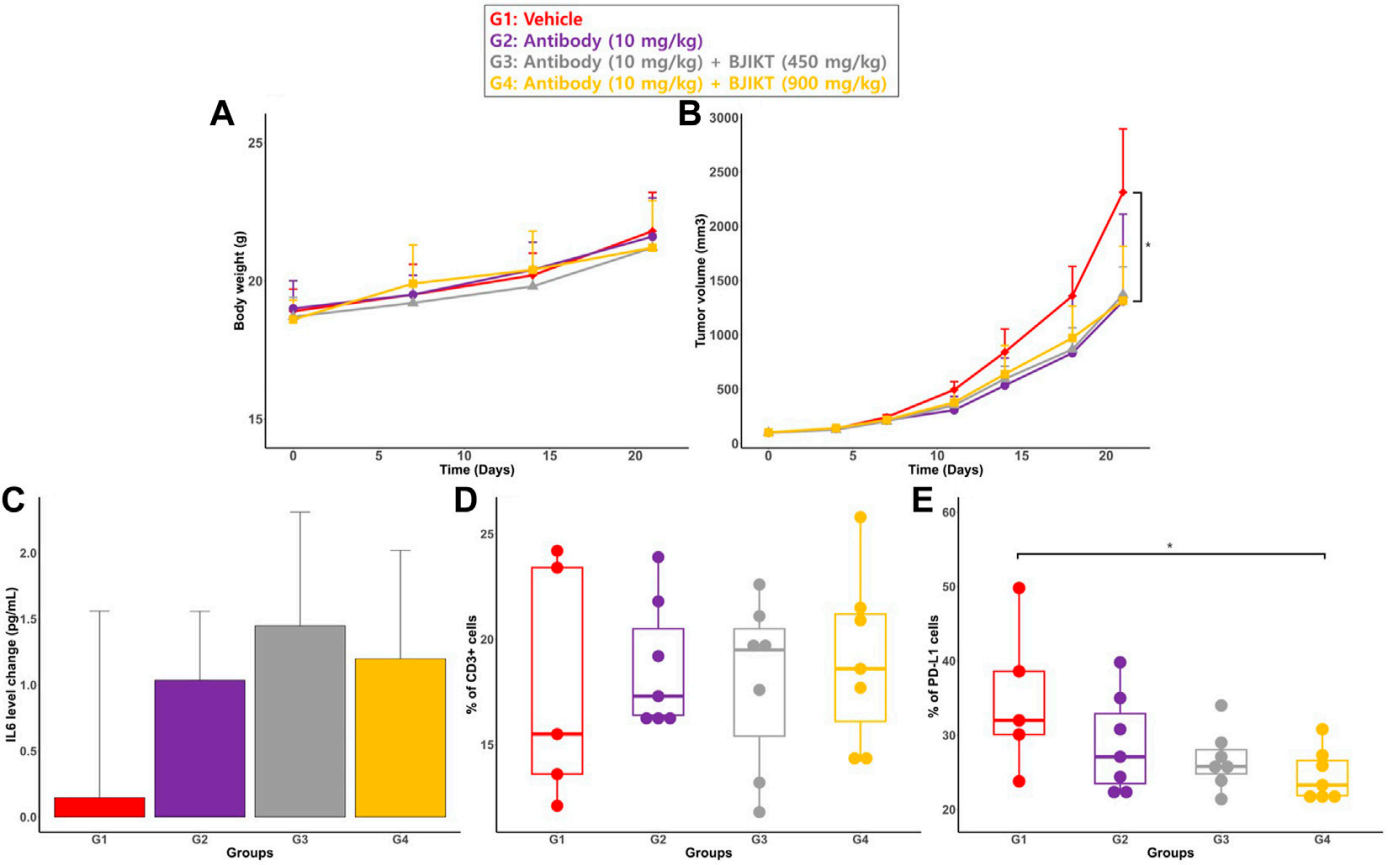
PD biomarkers for DDI evaluation of the combination therapy. **(A)** Body weight of mouse. Body weight of mouse were weighed every week (Day 0, 7, 14, and 21); **(B)** Tumor volumes. Tumor volumes were measured at specified days (Day 0, 4, 7, 11, 14, 18, and 21); **(C)** The change in IL-6 levels. The change in IL-6 levels is expressed as the change of IL-6 from the baseline (DAY 21–Day 0) **(D)** the percentage of CD3 cells on the tumor surface; **(E)** the percentage of positive PD-L1 on the tumor surface: * represents *p* < 0.05, compared to the control group.

The mice were monitored daily to check health status, and illness or abnormal behaviors were not observed. All animals survived until the end of the study period. All groups had no significant difference in body weight (Figure 2A).

### 3.2 Evaluation of pharmacokinetic drug interaction

**FIGURE 3:**
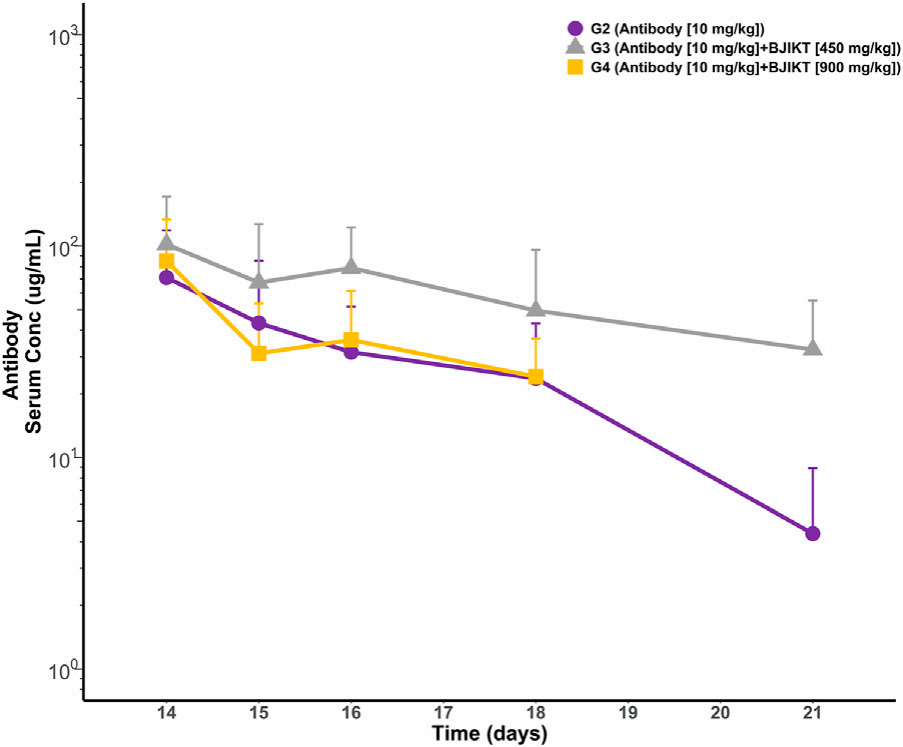
PK profiles of anti-PD-L1 antibody in mouse serum. Each point represents the mean and standard deviation.

In the pharmacokinetic drug interaction study, we considered that the anti-PD-L1 antibody may be unrelated to cytokine-modulation mechanisms since our study proved that peripheral cytokine levels in mice were not different between G1 (the saline group) and G2 (the anti-PD-L1 antibody monotherapy group). If the antibody is unrelated to cytokine-inflammation mechanisms, BJIKT might affect therapeutic protein clearance by altering immunogenicity or target-mediated disposition. To investigate the impact of BJIKT on the antibody PK, serum concentrations of the anti-PD-L1 antibody over time were measured using the developed ELISA method. The PK parameters of the anti-PD-L1 anti-body were determined using non-compartmental analysis (NCA) and compared between the anti-PD-L1 antibody monotherapy and combination therapy test groups. PK profiles of the antibody and PK parameters for the groups are shown in Figure 3. The mean ± standard deviation PK parameters of the antibody for these test groups are summarized in Table 1. The area under the curve from zero to the last measurement (AUCt) (mean ± SD) of the antibody for G2∼4 were 194.5 ± 146.4, 398.6 ± 299.2, and 177.6 ± 83.6 g/mL/day, respectively. The half-lives of the antibody for G2∼4 were 2.08 ± 1.16, 5.83 ± 4.56, and 3.02 ± 1.66 day, respectively. The clearance of the antibody for G2∼4 were 1.21 ± 0.48, 0.48 ± 0.24, and 0.72 ± 0.24 mL/day, respectively. The volume of distribution of the antibody for G2∼4 were 3.91 ± 2.74, 3.81 ± 3.67, and 3.57 ± 1.80 mL, respectively. NCA results showed that there were no significant differences in antibody PK parameters between the test groups.

**TABLE 1:** Pharmacokinetic parameters of anti-PD-L1 antibody for the test groups.

PK parameters	G2	G3	G4	*p*-val
*AUCt* (μg/mL·day)	194.51 ± 146.43	398.63 ± 299.22	177.65 ± 83.68	>0.05
Half-life (day)	2.08 ± 1.16	5.83 ± 4.56	3.02 ± 1.66	>0.05
*CL* (mL/day)	1.21 ± 0.48	0.48 ± 0.24	0.72 ± 0.24	>0.05
*Vd* (mL)	3.91 ± 2.74	3.81 ± 3.67	3.57 ± 1.80	>0.05

### 3.3 Evaluation of pharmacodynamic drug interaction

We evaluated pharmacodynamic drug interactions by measuring variable biomarkers related to pharmacological effects and immune responses. For pharmacodynamic interactions, 1) tumor volume growth, 2) serum cytokine level, and 3) tumor-infiltrating cells, and PD-L1 expression on the surface of tumor cells were measured and compared between all groups.

#### 3.3.1 Tumor volume growth

Tumor volumes at the end of the study (Day 21) were compared between all groups (Figure 2B). There was no significant difference in tumor volume at Day 21 between the test groups. While there was a non-significant trend for combination therapy to yield a higher antitumor response than monotherapy, each test group had significantly smaller tumor volumes than the control group (<0.05).

#### 3.3.2 Serum cytokine levels

Among the 5 cytokines, IL-6 was the only cytokine detected in mouse serum. Concentrations of other cytokines were under the method’s quantitative limit (Supplementary Figure S3 in Supplementary Material). To measure the change in cytokine levels during the treatment period, serum samples were analyzed at the study’s initial date/end date (Day 0/Day 21). The change in cytokine levels was expressed as the change in IL-6 levels from the baseline (DAY 21–Day 0) (Figure 2C). Statistical analysis showed that there was no significant difference in the change in IL-6 serum levels among all groups.

#### 3.3.3 Tumor infiltrating lymphocytes and PD-L1 expression

Values of CD3^+^ and PD-L1+ cells on tumor surface for all groups are described in Figures 2D, E, respectivlely. The cell percentage was measured using flow cytometry analysis. The results showed that the expression of CD3^+^ on tumor cells was not significantly different between all groups. Furthermore, there was no significant difference in the percentage of PD-L1+ on the surface of tumor cells between all groups. However, the percentage of PD-L1 in G4 is significantly lower than that of G1 (<0.05).

## 4 Discussion

The current study evaluated the PK-PD DDI potential between the anti-PD-L1 anti-body and BJIKT using the murine mouse model. In this study, murine lung cancer cells (CMT-167) were used to produce preclinical DDI data, which could predict human clinical DDI trials with NSCLC patients. A bioanalytical assay was developed and verified to determine the concentrations of the investigated drugs (anti-PD-L1 antibody and BJIKT) and applied to PK studies. PK DDI evaluation demonstrated that BJIKT does not affect the PK of the anti-PD-L1 antibody. Regarding the assessment of PD DDI, no antagonistic interactions or adverse immune responses were observed when the anti-PD-L1 antibody was co-administered with BJIKT.

The FDA has released draft guidance containing suggestions for the investigation of potential DDI for TP. The guidance identified two mechanisms of potential DDI that should be considered when DDI evaluation for TP is performed. The first DDI mechanism arises where the TP is a pro-inflammatory cytokine or acts as a cytokine modulator. For the mechanism in which the TP is a cytokine modulator, it would have indirect drug interactions with co-administered drugs as a perpetrator by regulating the expression of CYP enzymes (Tinel et al., 1995; Liptrott et al., 2009; Kenny et al., 2013). The second mechanism for DDI occurs where the TP is not related to cytokine modulators. Our study considered both DDI mechanisms and conducted associated analyses. As suggested in the FDA guidance related to DDI for TP, we tried to identify whether the anti-PD-L1 antibody acts as a cytokine modulator by measuring the peripheral cytokine profiles. As the change in the IL-6 levels in serum samples during the treatment period was not significantly different between all groups, the antibody is unrelated to cytokine modulation. From these findings, we concluded that atezolizumab would not affect the pharmacokinetic-related exposure of BJIKT. Hence, we focused on the effects of BJIKT on atezolizumab’s PK/PD changes and found no significant differences in PK-PD parameters between the test groups, indicating the absence of adverse herb-drug interaction for the combination therapy in the lung cancer mouse model.

BJIKT has been used to treat many diseases (Onogi et al., 2006; Tohda et al., 2008; Jeong et al., 2010; Yang et al., 2015). Given its immunomodulatory effects, recent research has investigated the potential of BJIKT, co-administered with western medicines, as an alternative therapy to treat a wide range of diseases (Ohgitani et al., 2014; Sato et al., 2015; Lee et al., 2021). PD-L1 expression in a tumor at pre-treatment is considered a predictive biomarker for ICI therapies (Davis and Patel, 2019). However, as a prognostic biomarker, higher PD-L1 expression was associated with poor clinical outcome in patients with NSCLC (Tuminello et al., 2020). Co-administration of BJIKT with the anti-PD-L1 antibody leads to decreased positive PD-L1 on the tumor surface at post-treatment with each dose increment of BJIKT (from 28.8% to 24.6% between G3 and G5). Based on these findings, BJIKT-induced inhibition of PD-L1 could lead to favorable therapeutic responses in combined therapy with atezolizumab.

Several further steps remain to clarify these findings. As extrapolation of animal data to clinical assessment of DDI for TP remains challenging, clinical DDI studies need to be conducted to confirm our results. With the issuance of the final FDA guidance released later, assessment of DDI on new primary PD biomarkers is recommended to augment our current results. Furthermore, exposure of Ginsenoside Rb1 in BJIKT changed when BJIKT was co-administered with atezolizumab (Supplementary Table S3 in Supplementary Material). Even though Ginsenoside Rb1 is not the only active compound among BJIKT ingredients and our study focused on the effects of BJIKT on the exposure or efficacy of atezolizumab, a BJIKT-related DDI mechanism study may also be warranted.

## 5 Conclusion

The current study investigated the potential DDI between BJIKT and the anti-PD-L1 antibody to illuminate the safety and efficacy of combination therapy using syngeneic mouse model. DDI evaluation was conducted following the suggestions from the FDA draft guidance of DDI for TP. To the best of our knowledge, this is the first study investigating DDI for a monoclonal antibody and herbal medicine, focusing on assessing both pharmacokinetic and pharmacodynamic DDI in the mouse model. With our approach, the researchers could apply other DDI cases between TP and herbal medicine. Further clinical studies are warranted to evaluate our results.

## Data Availability

The original contributions presented in the study are included in the article/Supplementary Materials, further inquiries can be directed to the corresponding authors.
