# Supplementary material for: Evaluation of the potential herb-drug interaction between Bojungikki-tang and PD-L1 immunotherapy in a syngeneic mouse model
Source: Front Pharmacol. 2023 May 18;14:1181263. doi: 10.3389/fphar.2023.1181263 (PMC10232755; doi:10.3389/fphar.2023.1181263)
Supplement: Supplementary file 1 [file DataSheet1.docx]

**Evaluation of the potential herb-drug interactions between Bojungikki-tang and PD-L1 immunotherapy**

**Supplementary document**

Sung-yoon Yang ^1†^, Jin-Mu Yi ^2†^, Jaemoo Chun ^2^, Seongwon Park ^1^, Tham Thi Bui ^1^, Hwi-yeol Yun ^1^, Jung-woo Chae ^1,^*, Mi-Kyung Jeong ^2,^*

^1^ College of Pharmacy, Chungnam National University, Daejeon, Republic of Korea

^2^ KM Convergence Research Division, Korea Institute of Oriental Medicine, Daejeon, Republic of Korea

Corresponding author: Jung-woo Chae, college of Pharmacy, Chungnam Nation university, jwchae@cnu.ac.kr (J.-w.C.); Mi-Kyung Jeong, Korea Institute of Oriental Medicine, oiny2000@kiom.re.kr (M.-K.J.)

**Table of contents**

[1. Fingerprint analysis of Bojungikki-tang 3](#_Toc112839259)

[1.1. LC-MS/MS chromatogram of Bojungikki-Tang (BJIKT) 3](#_Toc112839260)

[2. Development of ELISA assay for determination of anti-PD-L1 antibody in mouse serum 4](#_Toc112839261)

[2.1. Materials 4](#_Toc112839262)

[2.2. Development and validation of ELISA 4](#_Toc112839263)

[2.2.1. Development of ELISA 4](#_Toc112839264)

[2.2.2. Validation of ELISA 5](#_Toc112839265)

[2.3. Results 6](#_Toc112839266)

[2.3.1. Standard calibration curve 6](#_Toc112839267)

[2.3.2. Precision and accuracy 6](#_Toc112839268)

[3. Determination of serum cytokine levels 15](#_Toc112839269)

[3.1. Materials 15](#_Toc112839270)

[3.2. Sample preparation 15](#_Toc112839271)

[3.2.1. Preparation of standard samples 15](#_Toc112839272)

[3.2.2. Preparation of experimental samples 15](#_Toc112839273)

[3.2.3. Preparation of couple beads 15](#_Toc112839274)

[3.3. Cytokine assay 15](#_Toc112839275)

[3.4. Results 15](#_Toc112839276)

[4. Development of LC-MS/MS method for determination of active compounds for Bojungikki-tang in mice serum and its application to pharmacokinetic drug-drug interaction study 18](#_Toc112839277)

[4.1. The effects of anti-PD-L1 antibody on the pharmacokinetics of BJIKT 18](#_Toc112839278)

[4.2. LC-MS/MS method 19](#_Toc112839279)

[4.3. Results 19](#_Toc112839280)

# Fingerprint analysis of Bojungikki-tang

## LC-MS/MS chromatogram of Bojungikki-Tang (BJIKT)


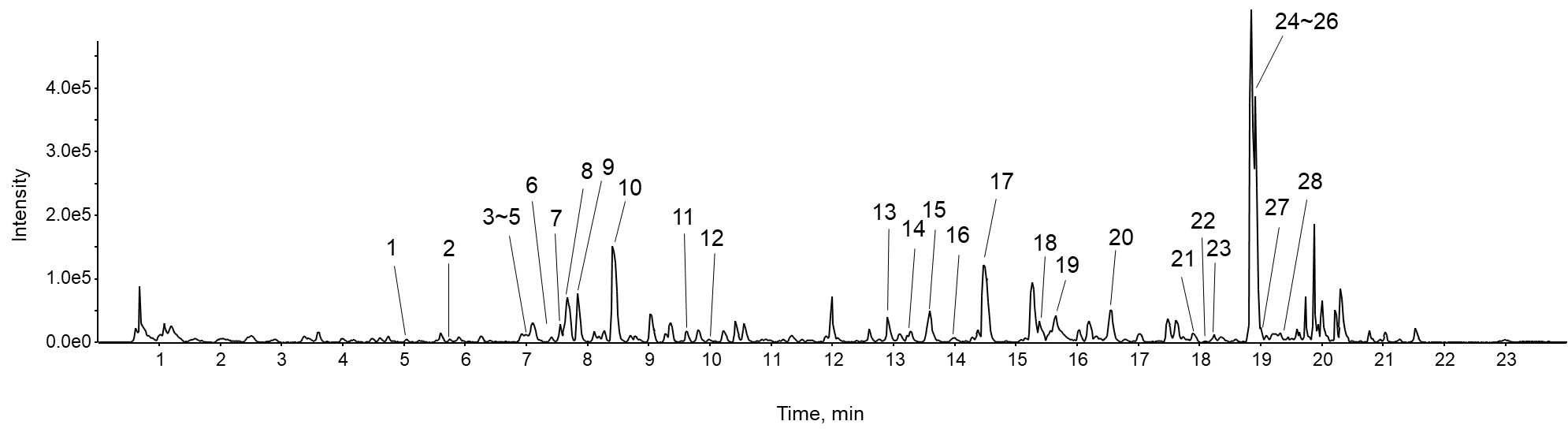


Figure S1. The representative base peak chromatogram of BJIKT extract. The analytical method is described in the published research paper using the same BJIKT extract. This chromatogram is modified and quoted from a published paper by Chun et al (Frontiers in Pharmacology 2022, 13, doi:10.3389/fphar.2022.901563.).

| **No.** | **Compound** | **Molecular formula** | **RT (min)** | **Content (mg/g)** | **No.** | **Compound** | **Molecular formula** | **RT (min)** | **Content (mg/g)** |
| --- | --- | --- | --- | --- | --- | --- | --- | --- | --- |
| 1 | caffeic acid | C_9_H_8_O_4_ | 5.10 | 0.0273 | 15 | formononetin | C_16_H_12_O_4_ | 13.55 | 0.0349 |
| 2 | magnoflorine | C_20_H_24_NO_4_ | 5.74 | 0.0105 | 16 | astragaloside IV | C_41_H_68_O_14_ | 14.02 | 0.0180 |
| 3 | ferulic acid | C_10_H_10_O_4_ | 7.07 | 0.1268 | 17 | glycyrrhizin | C_42_H_62_O_16_ | 14.49 | 5.6550 |
| 4 | liquiritin apioside | C_26_H_30_O_13_ | 7.10 | 1.8770 | 18 | saikosaponin A | C_42_H_68_O_13_ | 15.42 | 0.5603 |
| 5 | liquiritin | C_21_H_22_O_9_ | 7.14 | 0.4866 | 19 | 6-gingerol | C_17_H_26_O_4_ | 15.62 | 0.1073 |
| 6 | isoferulic acid | C_10_H_10_O_4_ | 7.41 | 0.1504 | 20 | atractylenolide III | C_15_H_20_O_3_ | 16.51 | 0.0934 |
| 7 | cimifugin | C_16_H_18_O_6_ | 7.56 | 0.0669 | 21 | astragaloside I | C_45_H_72_O_16_ | 17.81 | 0.0605 |
| 8 | nodakenin | C_20_H_24_O_9_ | 7.68 | 1.9347 | 22 | saikosaponin D | C_42_H_68_O_13_ | 18.06 | 0.0044 |
| 9 | narirutin | C_27_H_32_O_14_ | 7.84 | 2.6557 | 23 | ginsenoside Rg3 | C_42_H_72_O_13_ | 18.24 | 0.1029 |
| 10 | hesperidin | C_28_H_34_O_15_ | 8.43 | 7.2197 | 24 | decursin | C_19_H_20_O_5_ | 18.87 | 1.7510 |
| 11 | ginsenoside Rg1 | C_42_H_72_O_14_ | 9.62 | 0.3464 | 25 | decursinol angelate | C_19_H_20_O_5_ | 18.91 | 1.7510 |
| 12 | liquiritigenin | C_15_H_12_O_4_ | 10.01 | 0.0264 | 26 | atractylenolide II | C_15_H_20_O_2_ | 18.88 | 0.0029 |
| 13 | ginsenoside Rb1 | C_54_H_92_O_23_ | 12.91 | 0.7744 | 27 | 6-shogaol | C_17_H_24_O_3_ | 19.01 | 0.0061 |
| 14 | saikosaponin C | C_48_H_78_O_17_ | 13.27 | 0.1391 | 28 | atractylenolide I | C_15_H_18_O_2_ | 19.42 | 0.0020 |

Table S1. Quantitative analysis of chemical components of BJIKT (modified and quoted from a published paper by Chun et al, Frontiers in Pharmacology 2022, 13, doi:10.3389/fphar.2022.901563.).

# Development of ELISA assay for determination of anti-PD-L1 antibody in mouse serum

## Materials

Recombinant mouse PD-L1/B7-H1-Fc Chimera Protein, CF were purchased from R&D Systems® (Minneapolis, MN, USA). Tris-buffered saline with 0.1% Tween® 20 detergent (TBST) was obtained from Bethyl Laboratories®. Bovine serum albumin (BSA), Streptavidin-Peroxidase Polymer, and Ultrasensitive (1 mg/mL) were purchased from Sigma Aldrich® (St. Louis, MO, USA). SpectraMax Pardigm Multi-Mode Microplate Reader was obtained from Molecular devices (San Jose, USA). ElISA TMB substrate set was purchased from BioLegend. Stop solution was obtained from Komabiotech. Goat anti-Rat IgG (H+L) secondary antibody and biotin were purchased from Thermo scientific® (Waltham, MA, USA). Anti-PD-L1-mIgG1e3 InvivoFit™ (anti-PD-L1 antibody) was obtained from Invivogen (San Diego, CA, USA).

## Development and validation of ELISA

### Development of ELISA

#### Stock solution preparation

Two stock solutions (400 and 40 ug/mL) of Anti-PD-L1 antibody were prepared using 1X PBS solution

#### Matrix buffer preparation for ELISA

Blood samples were collected from drug-free C57BL/6 mice in serum separate tubes and centrifuged (4 °C, 4000 rpm, 10 min) to obtain the serum, kept in -80 °C until use for analysis. Matrix buffer (MB) was prepared using the blank serum diluted 1:1000 and 1:100 (v/v.), in incubation buffer (IB; 0.1 % BSA in TBST). The MB was spiked with different concentrations of anti-PD-L antibody

#### Standard and quality control sample preparation

Two sets of 400 ng/mL of anti-PD-L1 working solutions for ELISA were prepared using two stock solutions (400 and 40 ug/mL) diluted in MB (1:1000 and 1:100 [v:v]). Standard samples were prepared by serial dilution of the anti-PD-L1 working solutions (400 ng/mL) for ELISA in MB to establish the corresponding calibration standard curves. Quality control (QC) samples were also prepared by serial dilution of the anti-PD-L1 working solutions (400 ng/mL) for ELISA in MB.

#### Anti-PD-L1 antibody ELISA

A flat-bottom 96-well microplate was coated with 2 ug/mL of recombinant mouse-PD-L1 capture fusion protein and incubated 4 °C overnight. Next, the plate, washed five times with 250 uL/well of TBST, was blocked with 250 uL/well of IB for 1 h at room temperature to avoid unspecific reactions. Then, the plate was washed five times using 250 uL/well of TBST, and the standards, QC samples, and experimental samples (100 uL/well) were added and incubated for 2 hr at room temperature. For the labeling, the plate was washed five times using 250 uL/well of TBST and treated for 1 hr with a secondary antibody (diluted 1:100,000 [v/v] in IB). Then, the plate was washed five times using 250 uL/well of TBST. To conjugate Biotin-HRP, Streptavidin-peroxide (diluted 1:5000, [v/v] in IB) was added and incubated for 1hr at room temperature. The plate was washed five times using 250 uL/well of TBST and revealed with 100 uL/well of ELISA TMB for 10 min at the dark site. The reaction procedure was stopped by adding 100 uL/well of stop solution. The optical density was read at 450 nm in SpectraMax Pardigm Multi-Mode Microplate Reader using analytical software (SortMax^®^Pro, Molecular Device, San Jose, USA) to obtain STD curve, determine the concentrations for QC samples and experimental samples.

#### Standard calibration curve

The calibration curves were established using 4-parameter logistic. The parameters corresponding to the curved line (y= $D+\frac{A-D}{1+ \left( \frac{x}{C} \right)^{B}}$; where y, represents signal and x, the concentration of anti-PD-L1 antibody) were calculated for each assay

### Validation of ELISA

Partial validation process was performed in accordant with the recommendations of bioanalytical method validation guidance for industry to evaluate the following parameters: accuracy and precision

#### Standard calibration curve

The parameters corresponding to the curved line (A, B, C, and D) were calculated for each assay and statistically analyzed according to the intra-/inter-day variability. The standard criterium of the correlation parameter (R^2^) for the curve line was set > 0.99)

#### Precision and accuracy

To determine the precision and accuracy, 4 different concentration levels of QC samples (6.5, 19.5, 150, and 300 ng/mL) were prepared in triplicate and analyzed on the intra-/inter- day. The acceptable criteria for precision should be ≤ 20% (LLOQ: ≤25%) and accuracy should be in range of 80-120 % (LLoQ: 75-125%) according to FDA guidance.

## Results

### Standard calibration curve

Several standard calibration curves were successfully established with the range of 6.25 – 400 ng/mL with R^2^ > 0.99 (Figure S2). Table S2 lists the individual standard equations along with the correlation parameter.

### Precision and accuracy

The analyzed concentrations of QC samples were accordant with the acceptance criteria for precision and accuracy (accuracy: 80-120% [LLoQ: 75-125%], precision: ≤20% [LLoQ: ≤25%]). Table S2 lists individual analyzed concentrations with precision and accuracy.


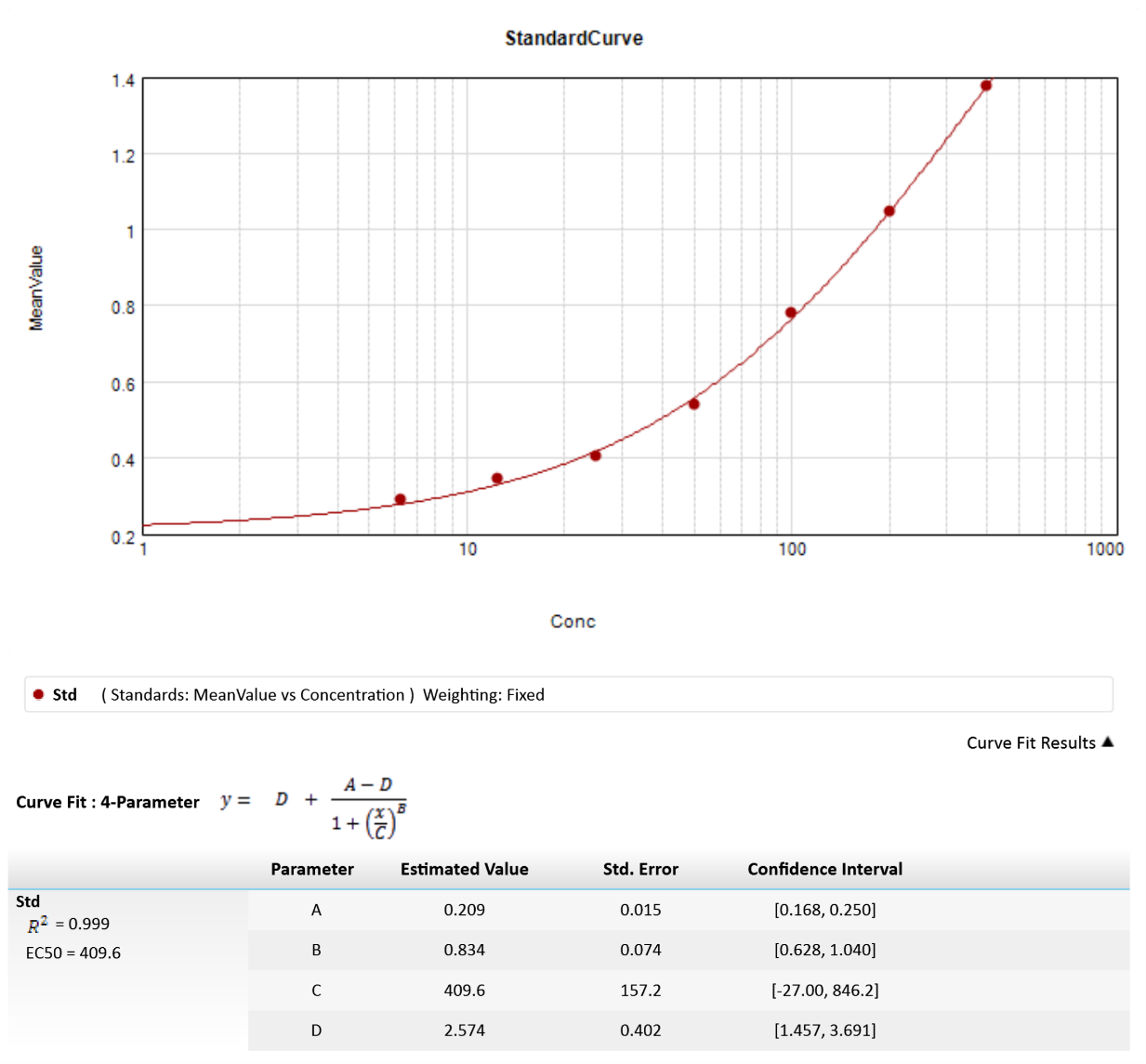


Figure S2-1 Standard calibration curve for Batch #2 [2021-10-16] (1:1000 dilution)

**
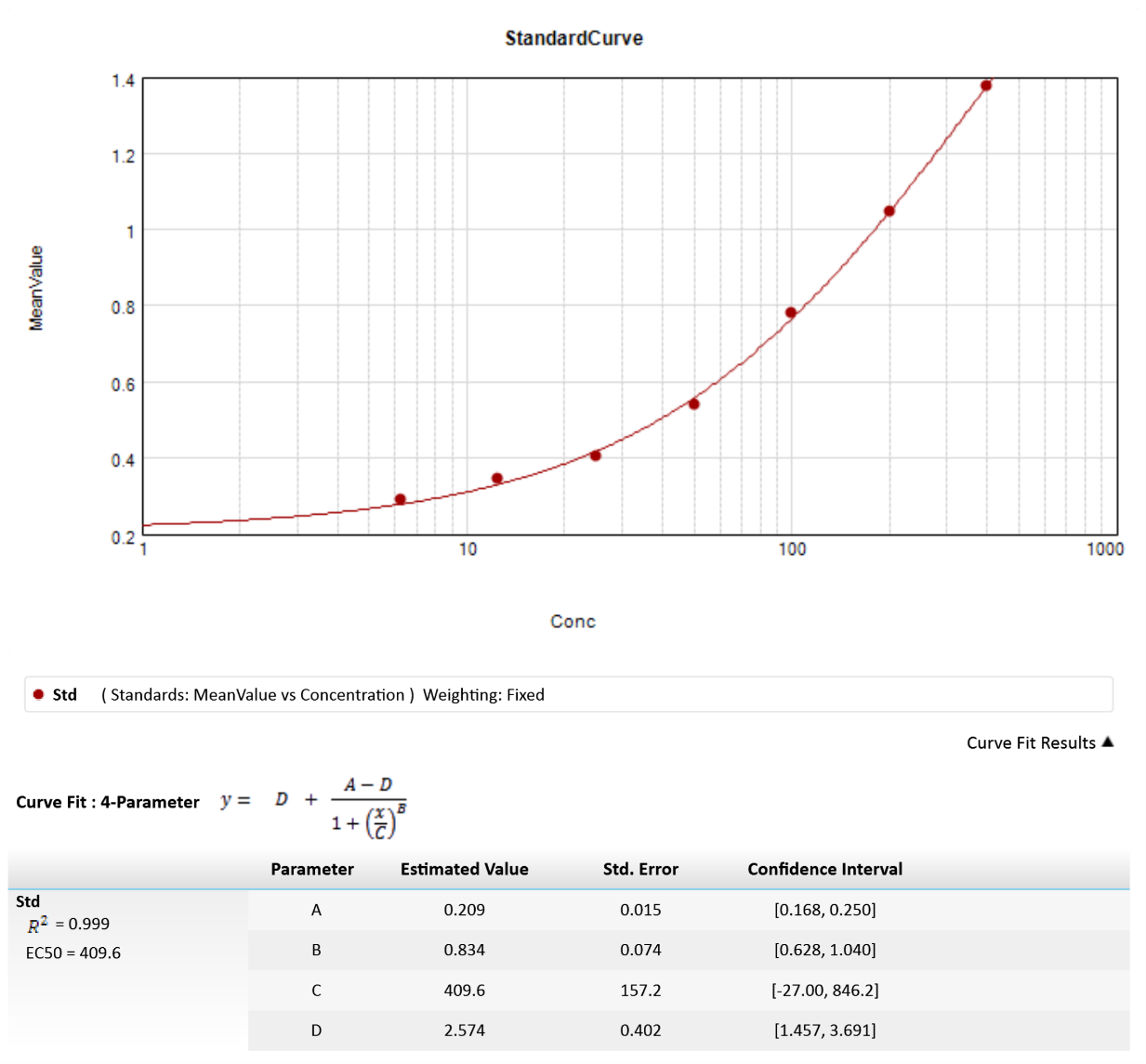
**

Figure S2-2 Standard calibration curve for Batch #3 [2021-10-17] (1:1000 dilution)


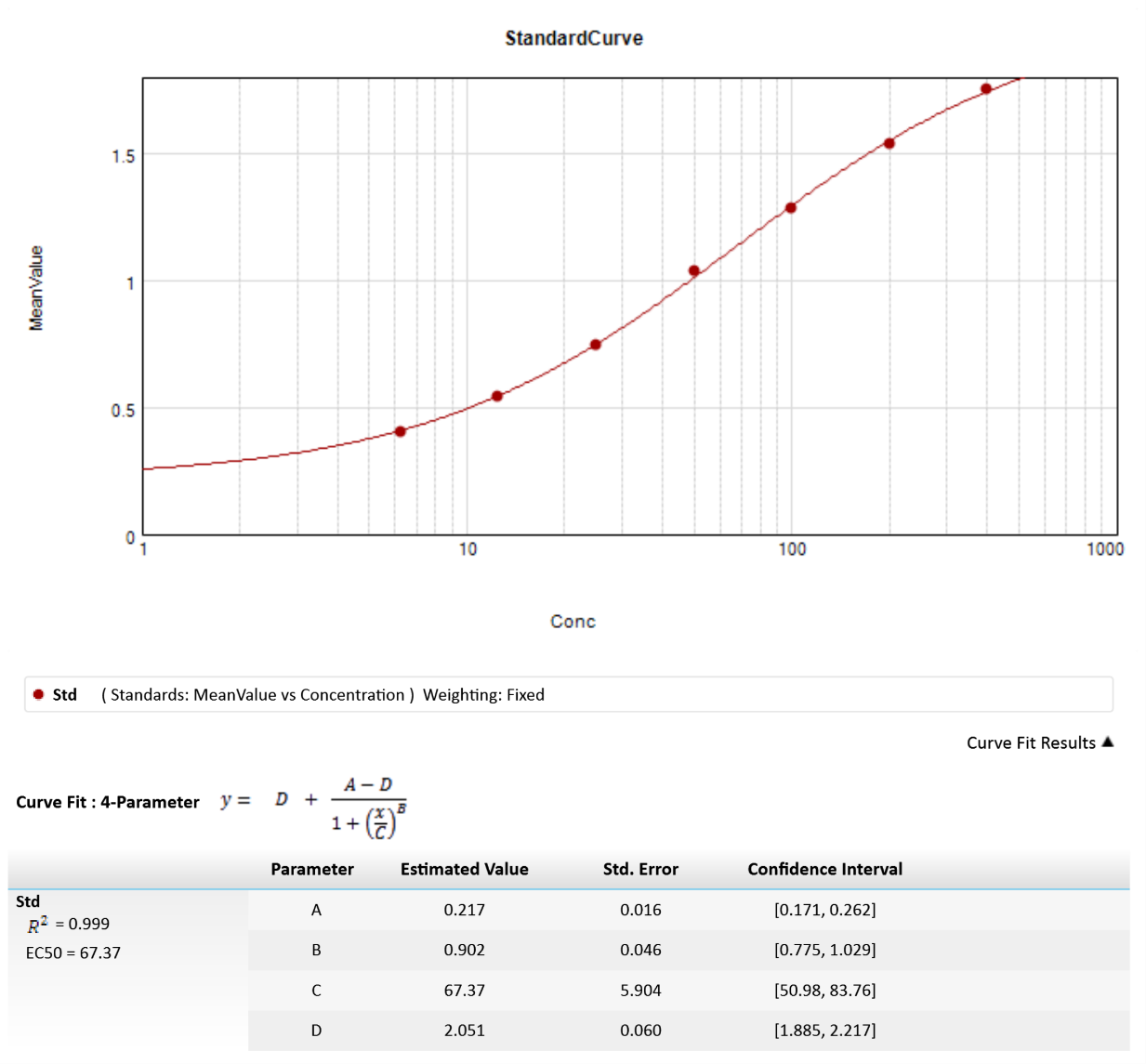


Figure S2-3 Standard calibration curve for Batch #4 [2021-10-18] (1:1000 dilution)

**
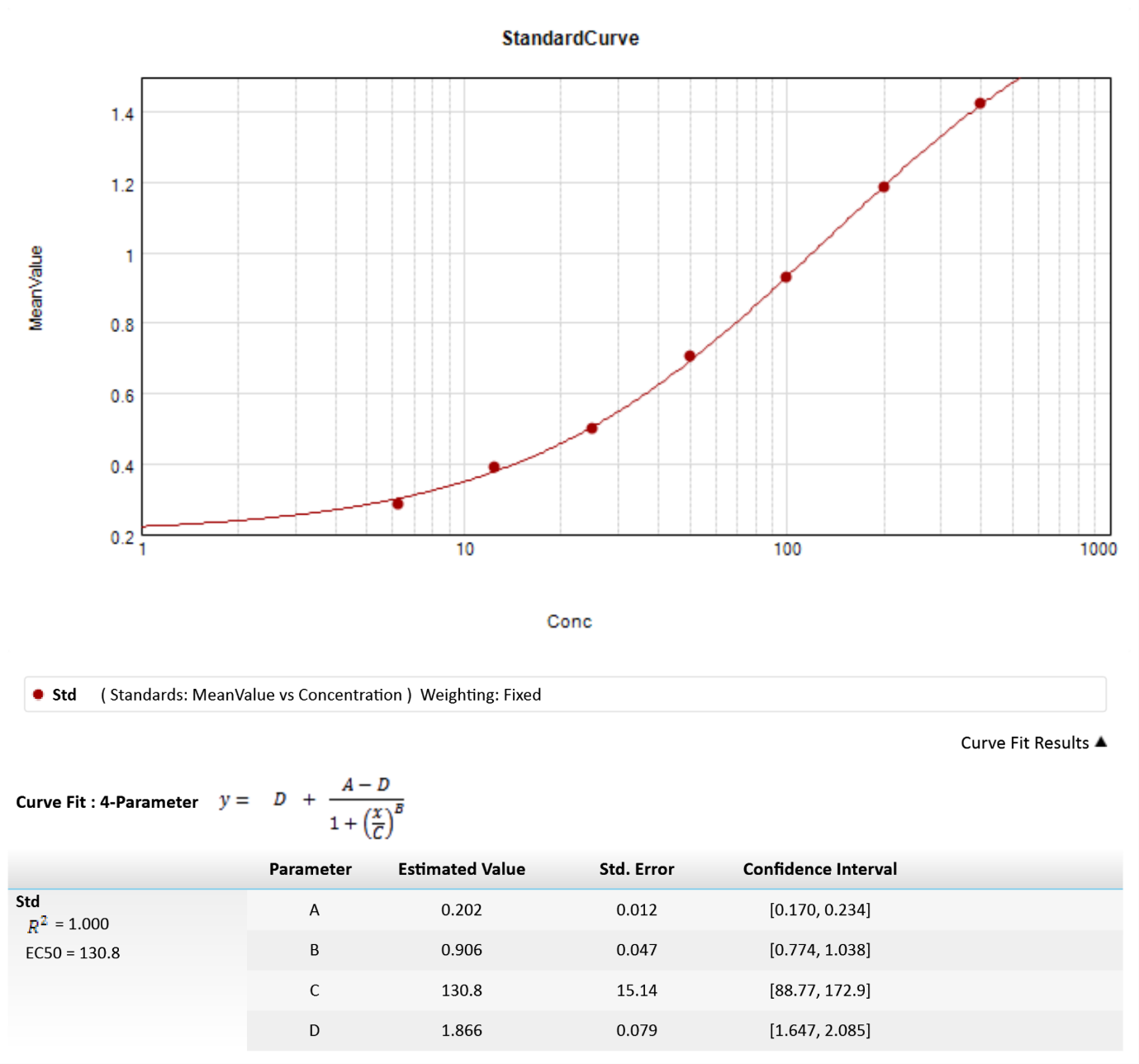
**

Figure S2-4 Standard calibration curve for Batch #1 [2021-10-19] (1:100 dilution)

**
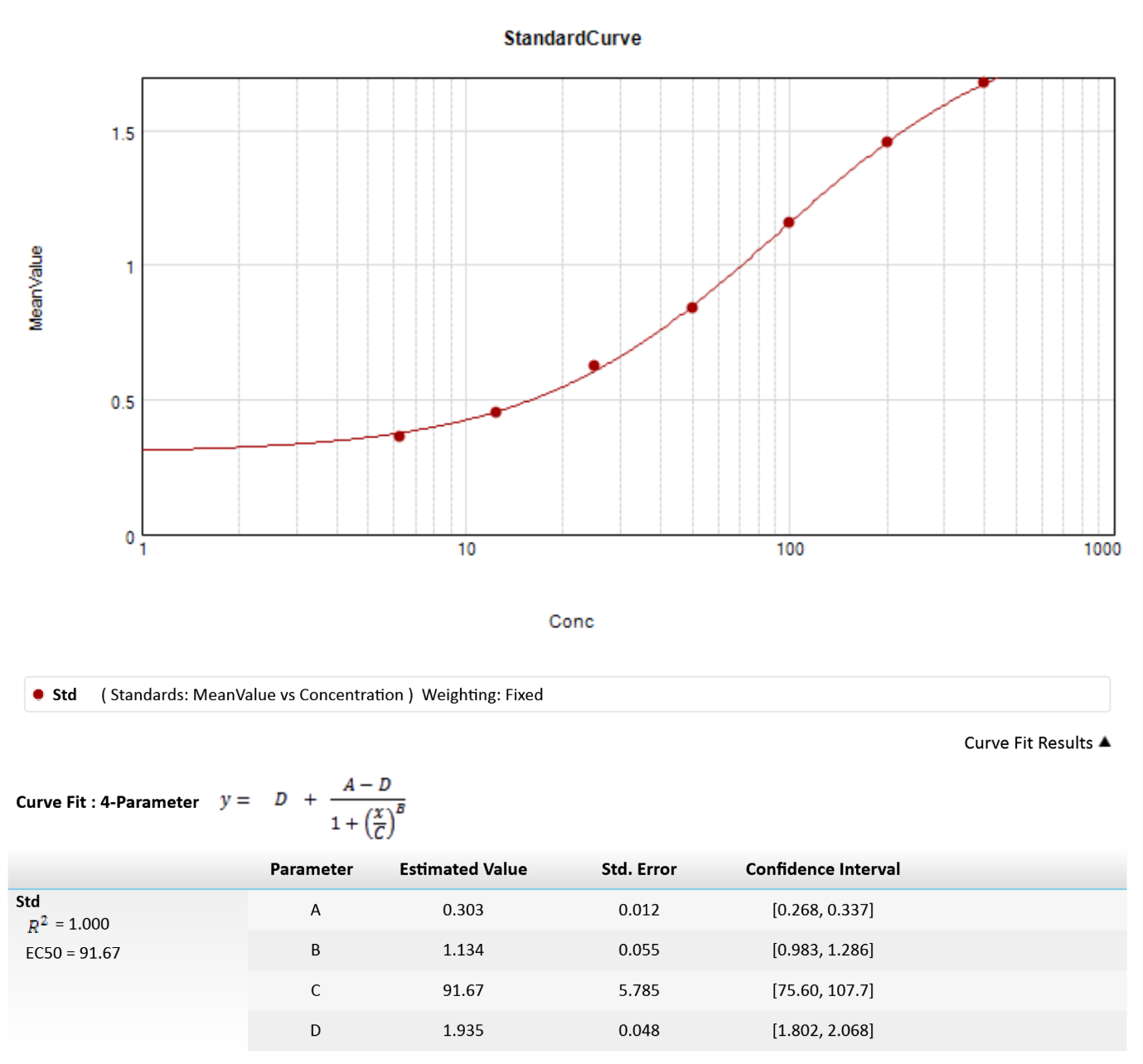
**

Figure S2-5 Standard calibration curve for Batch #2 [2021-10-20] (1:100 dilution)

**
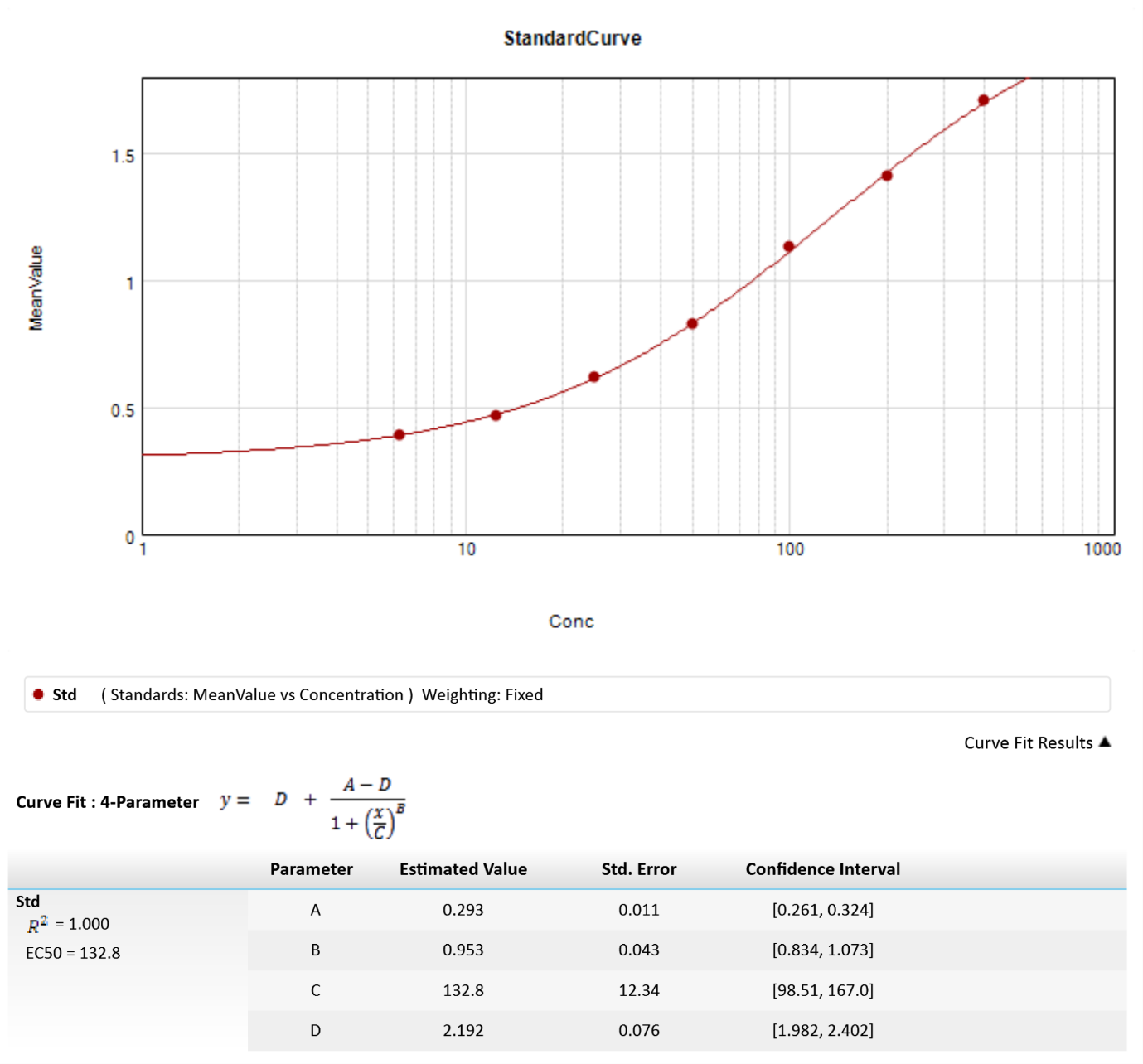
**

Figure S2-6 Standard calibration curve for Batch #3 [2021-10-21] (1:100 dilution)

Table S2

Table S2-1 Results of calibration curve (1:1000 dilution)

|  | | | | | | | | |
| --- | --- | --- | --- | --- | --- | --- | --- | --- |
| Batch | | Concentration for STD (ng/mL) | | | | | | |
| Date | Batch NO. | 6.25 | 12.5 | 25 | 50 | 100 | 200 | 400 |
| 2021.10.16 | #2 | 7.64 | 14.22 | 23.10 | 46.67 | 104.36 | 199.42 | 399.41 |
| Accuracy (%) | | 122.2 | 122.20 | 113.72 | 92.39 | 93.35 | 104.36 | 99.71 |
| 2021.10.17 | #3 | 6.11 | 12.47 | 25.12 | 49.74 | 104.13 | 184.82 | 461.80 |
| Accuracy (%) | | 97.816 | 97.82 | 99.78 | 100.48 | 99.47 | 104.13 | 92.41 |
| 2021.10.18 | #4 | 5.95 | 12.28 | 24.85 | 53.18 | 96.73 | 193.50 | 417.80 |
| Accuracy (%) | | 95.16 | 95.16 | 98.24 | 99.40 | 106.36 | 96.73 | 96.75 |
| Mean | | 6.566167 | 6.57 | 12.99 | 24.36 | 49.86 | 101.74 | 192.58 |
| SD | | 0.931507 | 0.93 | 1.07 | 1.10 | 3.26 | 4.34 | 7.34 |
| CV (%) | | 14.18647 | 14.19 | 8.21 | 4.51 | 6.53 | 4.27 | 3.81 |
| Accuracy (%) | | 105.0587 | 105.06 | 103.91 | 97.42 | 99.73 | 101.74 | 96.29 |

Table S2-2 Precision and accuracy (1:1000 dilution)

| Batch NO. | QC (ng/mL) | 1 | 2 | 3 | 4 | 5 | Within-run | | | |
| --- | --- | --- | --- | --- | --- | --- | --- | --- | --- | --- |
|  |  |  |  |  |  |  | Mean | SD | CV (%) | Accuracy (%) |
| #2 | 6.5 | 6.02 | 7.14 | 8.41 | 8.18 | - | 7.44 | 2.03 | 30.63 | 102.06 |
|  | 19.5 | 15.99 | 14.76 | 15.99 | 17.95 | - | 16.17 | 1.86 | 11.98 | 79.56 |
|  | 150 | 146.34 | 139.36 | 160.25 | 143.52 | - | 147.37 | 11.05 | 7.68 | 95.92 |
|  | 300 | 308.51 | 316.22 | 282.81 | 321.47 | - | 307.25 | 24.60 | 8.24 | 99.49 |
| #3 | 6.5 | 5.90 | 6.08 | 6.03 | 5.92 | - | 5.98 | 0.09 | 1.47 | 92.03 |
|  | 19.5 | 18.67 | 18.44 | 19.17 | 18.00 | - | 18.57 | 0.49 | 2.62 | 95.22 |
|  | 150 | 169.82 | 163.78 | 145.35 | 148.51 | - | 156.86 | 11.81 | 7.53 | 104.58 |
|  | 300 | 272.45 | 292.73 | 356.90 | 318.21 | - | 310.07 | 36.40 | 11.74 | 103.36 |
| #4 | 6.5 | 5.62 | 6.45 | 5.34 | 6.20 | 5.62 | 5.84 | 0.46 | 7.91 | 89.92 |
|  | 19.5 | 17.03 | 18.18 | 15.80 | 17.81 | 19.34 | 17.63 | 1.32 | 7.49 | 90.42 |
|  | 150 | 141.11 | 120.55 | 119.55 | 145.63 | 144.36 | 134.24 | 13.06 | 9.73 | 89.49 |
|  | 300 | 208.74 | 241.27 | 234.06 | 255.40 | 300.89 | 248.07 | 34.03 | 13.72 | 82.69 |
| Between-run | 6.5 | | | | | | 6.15 | 0.42 | 6.85 | 94.67 |
|  | 19.5 | | | | | | 17.24 | 1.56 | 9.08 | 88.40 |
|  | 150 | | | | | | 145.00 | 11.35 | 7.83 | 96.66 |
|  | 300 | | | | | | 285.54 | 32.96 | 11.54 | 95.18 |

Table S2-3 Results of calibration curve (1:100 dilution)

| Batch | | Concentration for STD (ng/mL) | | | | | | |
| --- | --- | --- | --- | --- | --- | --- | --- | --- |
| Date | Batch NO. | 6.25 | 12.5 | 25 | 50 | 100 | 200 | 400 |
| 2021.10.19 | #1 | 5.12 | 13.44 | 24.31 | 51.70 | 99.66 | 196.51 | 414.57 |
| Accuracy (%) | | 81.98 | 107.53 | 97.26 | 103.41 | 99.66 | 98.26 | 103.64 |
| 2021.10.20 | #2 | 5.08 | 12.13 | 26.75 | 48.88 | 100.76 | 200.39 | 402.17 |
| Accuracy (%) | | 81.35 | 97.06 | 107.02 | 97.76 | 100.76 | 100.19 | 100.54 |
| 2021.10.20 | #3 | 6.08 | 12.07 | 25.41 | 49.42 | 103.51 | 192.76 | 406.93 |
| Accuracy (%) | | 97.27 | 96.52 | 101.65 | 98.84 | 103.51 | 96.38 | 101.73 |
| Mean | | 5.43 | 12.55 | 25.49 | 50.00 | 101.31 | 196.55 | 407.89 |
| SD | | 0.56 | 0.78 | 1.22 | 1.50 | 1.98 | 3.81 | 6.26 |
| CV (%) | | 10.38 | 6.18 | 4.79 | 3.00 | 1.96 | 1.94 | 1.53 |
| Accuracy (%) | | 86.87 | 100.37 | 101.97 | 100.00 | 101.31 | 98.28 | 101.97 |

Table S2-4 Precision and accuracy (1:100 dilution)

| Batch NO. | QC (ng/mL) | 1 | 2 | 3 | 4 | 5 | Within-run | | | |
| --- | --- | --- | --- | --- | --- | --- | --- | --- | --- | --- |
|  |  |  |  |  |  |  | Mean | SD | CV (%) | Accuracy (%) |
| #1 | 6.5 | 5.52 | 6.37 | 5.41 | 5.73 | - | 5.76 | 0.43 | 7.46 | 88.61 |
|  | 19.5 | 18.07 | 16.55 | 16.07 | 16.08 | - | 16.69 | 0.95 | 5.68 | 85.61 |
|  | 150 | 134.08 | 130.42 | 123.04 | 126.61 | - | 128.54 | 4.77 | 3.71 | 85.69 |
|  | 300 | 252.71 | 236.33 | 240.75 | 238.75 | - | 242.13 | 7.28 | 3.01 | 80.71 |
| #2 | 6.5 | 5.75 | 5.19 | 5.30 | 4.33 | 4.49 | 5.01 | 0.59 | 11.79 | 77.06 |
|  | 19.5 | 17.49 | 15.08 | 17.35 | 16.12 | 18.47 | 16.90 | 1.32 | 7.80 | 86.67 |
|  | 150 | 125.81 | 116.19 | 147.51 | 118.26 | 146.63 | 130.88 | 15.21 | 11.62 | 87.25 |
|  | 300 | 254.80 | 245.94 | 268.15 | 263.60 | 368.78 | 280.25 | 50.22 | 17.92 | 93.42 |
| #3 | 6.5 | 4.77 | 5.85 | 5.20 | 6.01 | 4.88 | 5.34 | 0.56 | 10.49 | 82.18 |
|  | 19.5 | 16.58 | 17.27 | 17.48 | 17.52 | 18.09 | 17.39 | 0.55 | 3.15 | 89.16 |
|  | 150 | 143.85 | 149.39 | 156.99 | 147.92 | 151.28 | 149.88 | 4.82 | 3.22 | 99.92 |
|  | 300 | 277.09 | 280.49 | 277.42 | 292.79 | 315.85 | 288.73 | 16.46 | 5.70 | 96.24 |
| Between-run | 6.5 | | | | | | 5.37 | 0.38 | 7.00 | 82.62 |
|  | 19.5 | | | | | | 16.99 | 0.36 | 2.09 | 87.15 |
|  | 150 | | | | | | 136.43 | 11.71 | 8.58 | 90.96 |
|  | 300 | | | | | | 270.37 | 24.82 | 9.18 | 90.12 |

# Determination of serum cytokine levels

## Materials

Bio-Plex Pro Mouse Cytokine 5-plex Assay kits (assay, reagent, and diluent components) were purchased from BIO-RAD.

## Sample preparation

### Preparation of standard samples

Eight-point standard concentrations were prepared with a 4-fold dilution between each point using reconstituted standard vial from the kit to establish standard calibration curves.

### Preparation of experimental samples

Experimental samples were prepared using the collected serum samples diluent 1:4 (v/v.) in Bio-Plex sample diluent.

### Preparation of couple beads

96-well plates were added with Bio-Plex capture antibody/magnetic bead conjugates. Then standard samples, blank samples, experimental samples were added to plates. Next, Bio-Plex detection antibodies and Bio-Plex Fluorophore-Streptavidin conjugate were added to the plates.

## Cytokine assay

The optical density of was read in Bio-Plex 200 systems using Bio-Plex Manager^TM^ software to obtain STD curve and determine concentrations of the multiple serum cytokines (IFN-γ, TNF-α, IL-2, IL-6, IL-10) for experimental samples. The calibration curves were established using 5-parameter logistic. The parameters corresponding to the curved line (y= $A+\frac{B-A}{{1+ \left( \frac{x}{C} \right)^{-D}}^{E}}$; where y, represents signal and x, the concentration of cytokine). The parameters corresponding to the curved line (A, B, C, D, and E) were calculated for each assay

## Results

Several standard calibration curves for multiple cytokines were successfully established (Figure S3).

Figure S3-1 Standard calibration curve for IFN-γ.
[y= $\mathbf{0.0148628+}\frac{\mathbf{11328.4-0.0148628}}{{\mathbf{1+}\left( \frac{\mathbf{x}}{\mathbf{2511.15}} \right)^{\mathbf{-1.35583}}}^{\mathbf{0.769978}}}$] LLOQ and ULOQ were 0.79 and 18639.52 pg/mL, respectively

Figure S3-2 Standard calibration curve for IL-6.
[y= $\mathbf{13.5126+}\frac{\mathbf{18706.1-13.5126}}{{\mathbf{1+}\left( \frac{\mathbf{x}}{\mathbf{1611.76}} \right)^{\mathbf{-1.35583}}}^{\mathbf{0.769978}}}$] LLOQ and ULOQ were 0.74 and 11819.23 pg/mL, respectively

Figure S3-3 Standard calibration curve for TNF-α.
[y= $\mathbf{0.703609+}\frac{\mathbf{24248.1-0.703609}}{{\mathbf{1+}\left( \frac{\mathbf{x}}{\mathbf{17071.3}} \right)^{\mathbf{-1.11791}}}^{\mathbf{0.943818}}}$] LLOQ and ULOQ were 3.43 and 60607.69 pg/mL, respectively

Figure S3-4 Standard calibration curve for IL-10.
[y= $\mathbf{2.1064+}\frac{\mathbf{13512.3-2.1064}}{{\mathbf{1+}\left( \frac{\mathbf{x}}{\mathbf{7251.87}} \right)^{\mathbf{-4.56971}}}^{\mathbf{0.257381}}}$] LLOQ and ULOQ were 2.96 and 15450.32 pg/mL, respectively

Figure S3-4 Standard calibration curve for IL-10.
[y= $\mathbf{0.854912+}\frac{\mathbf{16347.1-0.854912}}{{\mathbf{1+}\left( \frac{\mathbf{x}}{\mathbf{2714.59}} \right)^{\mathbf{-10.5859}}}^{\mathbf{0.1}}}$] LLOQ and ULOQ were 1.21 and 5294.72 pg/mL, respectively

# Development of LC-MS/MS method for determination of active compounds for Bojungikki-tang in mice serum and its application to pharmacokinetic drug-drug interaction study

## The effects of anti-PD-L1 antibody on the pharmacokinetics of BJIKT

Since the metabolism underlying drug-drug interaction for therapeutic proteins remains unclear, anti-PD-L1 antibody could influence the pharmacokinetics of BJIKIT. Therefore, we evaluated the impacts of anti-PD-L1 antibody on the pharmacokinetics of BJIKT. In animal study, we allocated the female C57BL/6 mice (n=3) inoculated with CMT-167 cells, whose tumor volume reached 80 ~ 120 mm^3^, to the group 5 (G5) and gave 900 mg/kg of BJIKT once daily using oral route for consecutive 21 days. Ginsenoside Rg1 (Rg1) and ginsenoside Rb1 (Rb1) were selected as the investigated compounds of BJIKT for PK study based on the composition of BJIKT and literature. To evaluate the PK DDI, we measured the concentrations of Rb1 and Rg1 for G4 and G5, calculated the PK parameters using NCA, and conducted statistical analysis to compare their PK parameters. For the pharmacokinetic analysis of BJIKT, blood samples (80~100 uL) for G4 and G5 were collected into serum separating tube (SST) at the indicated time points 0.5, 1, 4, 8, 24 hr post-dose of BJIKT on Day 14.

## LC-MS/MS method

The concentrations of Rb1 and Rg1 in mouse serum were quantified by the fast and robust method using high performance liquid chromatography (1200 series HPLC; Agilent, Santa Clara, CA, USA) coupled to mass spectrometry (Qtrap 4000; Sciex, Framingham, MA, USA). The two compounds in were separated on XTERRA C18 reverse column (3.5 μm, 2.1 x 50 mm, Waters Milford, MA, USA) at a column temperature of 24 ℃. The mobile phase was composed of 0.1 % formic acid water (A) and methanol (B) at a flow rate of 0.4 mL/min. The gradient was as follows: 1 % B at 0~0.50 min, 1~ 90 % B at 0.50~2.40 min, 90 ~70 % B at 2.40~2.41, 70 % B at 2.41~4.00, 70~90 % B at 4.00~4.01, 90 % B at 4.01~6.00, 90~1 % B at 6.00~6.01, 1 % B at 6.01~11.00. The analysis was conducted with an electrospray ionization probe in the positive ion mode. The ion spray voltage was 5500 V, and the source temperature was 550 °C. Multiple reaction monitoring transitions of each analyte were as follows: m/z 1131.8 → 365.2 for Rb1, m/z 823.6 → 643.4 for Rg1, m/z 426.3 → 175.1 for domperidone (internal standard). Calibration standard samples were prepared to set up calibration range, and Quality control (QC) samples were run in triplicate to test accuracy of the developed method. Calibration curves for both compounds were linear in the range of 1.5 ~ 50 ng/mL (R^2^ > 0.99) and the mean concentration of QC samples were within 15 % of the nominal values. The obtained data were used to calculate PK parameters of active compounds of BJIKT.

## Results

Rb1 was detected in all PK samples, however Rg1 was below the limit of quantitation in the samples. PK parameters of Rb1 for G4 and G5 are shown in Table S3. The NCA results indicated that PK parameters were significantly different between G4 and G5, showing that PK characteristics of Rb1 were changed in the presence of anti-PD-L1 antibody. Given that pharmacological response was not significantly different in G1~G4, the decreased exposure of Rb1 in the presence of anti-PD-L1 is not associated with DDI-induced risk. Furthermore, there are many other pharmacologically active compounds in BJIKT other than Rb1. Therefore, further studies are needed to determine the pharmacokinetics of active compounds to help in predicting potential DDI.

Table S3 PK parameters of ginsenoside Rb1 for G4 and G5.

| **PK parameters** | **G4** | **G5** | **P-val** |
| --- | --- | --- | --- |
| *AUC* (ng/mL*hr) | 27.93 ± 6.92 | 156.12 ± 42.91 | < 0.05 |
| Cmax (ng/mL) | 5.01 ± 1.25 | 9.97 ± 2.12 | < 0.05 |
| Half-life (hr) | 6.08 ± 2.04 | 13.57 ± 1.04 | < 0.05 |
| *CL* (mL/hr/kg) | 11.54 ± 0.95 | 1.96 ± 0.33 | < 0.05 |
| *Vd* (mL/kg) | 104.02 ± 42.35 | 59.63 ± 23.67 | < 0.05 |
